# Effect of bioelectrical impedance technology on the prognosis of dialysis patients: a meta-analysis of randomized controlled trials

**DOI:** 10.1080/0886022X.2023.2203247

**Published:** 2023-05-03

**Authors:** Kaibi Yang, Shujun Pan, Nan Yang, Juan Wu, Yueming Liu, Qiang He

**Affiliations:** a Urology & Nephrology Center, Department of Nephrology, Zhejiang Provincial People’s Hospital (Affiliated People’s Hospital, Hangzhou Medical College), Hangzhou, Zhejiang, China; b Department of Nephrology, the First Affiliated Hospital of Zhejiang Chinese Medical University (Zhejiang Provincial Hospital of Traditional Chinese Medicine), Hangzhou, Zhejiang, China

**Keywords:** Dialysis, bioimpedance, fluid overload, prognosis

## Abstract

Managing patient ‘dry weight’ according to clinical standards has deficiencies. Research has focused on the effectiveness of using bioelectrical impedance technology for fluid management in dialysis patients. Whether bioelectrical impedance monitoring can improve dialysis patients prognoses remain controversial. We performed a meta-analysis of randomized controlled trials to determine whether bioelectrical impedance was effective in improving dialysis patients prognoses. The primary outcome was all-cause mortality (13.6 ± 9.1 months). Secondary outcomes were left ventricular mass index (LVMI), arterial stiffness assessed using Pulse Wave Velocity (PWV), and N-terminal brain natriuretic peptide precursor (NT-proBNP). Of 4,641 citations retrieved, we identified 15 eligible trials involving 2763 patients divided into experimental (*n* = 1386) and control (*n* = 1377) groups. In 14 studies with mortality data, the meta-analysis showed that bioelectrical impedance intervention reduced the risk of all-cause mortality (rate ratios [RR]: 0.71; 95% confidence interval [CI]: 0.51, 0.99; *p* = .05; I2 = 1%). Subgroup analysis of patients on hemodialysis (RR: 0.72; 95% CI: 0.42, 1.22; *p* = .22) and peritoneal dialysis (RR: 0.62; 95% CI: 0.35, 1.07; *p* = .08) showed no significant mortality difference between intervention and control groups. It reduced the risk of all-cause mortality in the Asian population (RR: 0.52; *p* = .02), and reduced NT-proBNP (mean difference [MD]: −1495.73; *p* = 0.002; *I*^2^=0%) and PWV (MD: −1.55; *p* = .01; *I*^2^=89%). Bioelectrical impedance intervention reduced the LVMI in hemodialysis patients (MD: −12.69; *p* < .0001; *I*^2^=0%). Our analysis shows that in dialysis patients, bioelectrical impedance technology intervention could reduce, but not eliminate, the risk of all-cause mortality. Overall, this technology can improve the prognosis of dialysis patients.

## Introduction

In patients requiring dialysis, volume overload is associated with hypertension and cardiac dysfunction and is a major risk factor for all-cause and cardiovascular mortality [1]. Previous studies have shown that in dialysis patients, cardiovascular disease is the main cause of death, accounting for more than 50% of the known causes of death, and their cardiovascular mortality is nine times that of the general population [2]. Left ventricular hypertrophy (LVH) is prevalent in dialysis patients, and individuals with LVH have a 2–4 times higher risk of cardiovascular events [3]. Furthermore, the body fluid load of dialysis patients eventually leads to heart failure [4], and fluid overload has a high predictive value for all-cause mortality in these patients [5]. In clinical practice, the dry weight of dialysis patients is usually assessed according to their clinical symptoms and signs [6]. However, this method is less reliable, as it is dependent on the subjective judgment of doctors. Therefore, methods that can objectively guide the fluid management of dialysis patients are being explored. In the last decade, lung ultrasound has been used to assess the volume load in dialysis patients [7]. A randomized trial of dialysis patients with a high cardiovascular risk showed that although guidance with lung ultrasound was more effective in reducing pulmonary edema when compared to guidance according to clinical criteria, it had no significant effect on all-cause mortality [8]. Another randomized trial using blood volume monitoring to determine the dry weight in dialysis patients showed increased mortality when compared with using traditional clinical standards [9].

Bioelectrical impedance monitoring may allow better management of the dry weight in dialysis patients than other techniques provide [10]. Bioelectrical impedance technology is currently divided into two types: bioelectrical impedance spectroscopy (BIS) and bioelectrical impedance analysis (BIA). Both types analyze the body’s resistance and reactance by measuring the current applied to distant electrodes on the body’s surface to estimate the composition of the body, including systemic water, extracellular water (ECW), and intracellular water (ICW) [11]. Bioelectrical impedance technology has proven to be a practical body fluid volume measurement tool. Moreover, a large retrospective trial revealed that chronic fluid overload assessed by bioelectrical impedance technology is an independent risk factor for patient mortality [12]. On the other hand, previously conducted trials have shown inconsistent results on whether bioelectrical impedance technology can reduce mortality in dialysis patients.

Therefore, we performed a meta-analysis of randomized controlled trials (RCTs) involving bioelectrical impedance technology as an intervention in dialysis patients to determine whether its clinical application improved their prognosis.

## Methods

### Retrieval strategy

**Figure 1.**
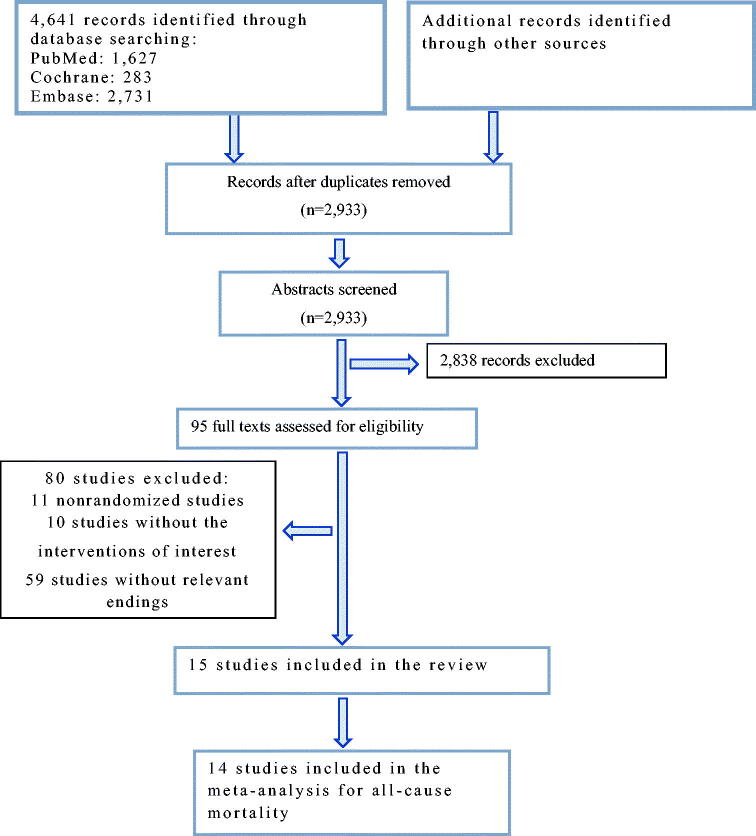
Flow diagram of included studies.

We conducted two searches using PubMed, Cochrane, and Embase databases for relevant English articles published up to March 18, 2022. In the first search, we used the English search terms ‘dialysis’ with ‘renal dialysis’ [Mesh] as the subject terms and ‘dialysis renal,’ ‘renal dialyzes,’ ‘dialyzes renal,’ ‘hemodialyses,’ ‘hemodialysis,’ ‘dialysis extracorporeal,’ and ‘dialyzes extracorporeal,’ as free words. In the second search, we search ‘bioelectrical impedance’ with ‘electric impedance’ [Mesh] as the subject terms, and ‘impedance electric,’ ‘electrical impedance,’ ‘impedance electrical,’ and ‘impedance,’ as free words. Complete details of the search strategy are presented in Figure 1 and Table 1. This meta-analysis was registered in PROSPERO (https://www.crd.york.ac.uk/PROSPERO/, number: CRD42022330022).

**Table 1.** Retrieval strategy.

Pubmed	‘Renal Dialysis’[Mesh] dialyses renal [Title/Abstract] renal dialyses [Title/Abstract] dialysis renal [Title/Abstract] hemodialyses [Title/Abstract] haemofiltration [Title/Abstract] hemodiafiltration [Title/Abstract] peritoneal dialysis [Title/Abstract] extracorporeal dialysis [Title/Abstract] extracorporeal dialyses [Title/Abstract] dialyses extracorporeal [Title/Abstract] hemodialysis [Title/Abstract] dialysis extracorporeal [Title/Abstract]	‘Electric Impedance’[Mesh] bioelectrical impedance [Title/Abstract] resistance electrical [Title/Abstract] impedance [Title/Abstract] impedance electric [Title/Abstract] impedance electrical [Title/Abstract] bioimpedance [Title/Abstract] impedance cardiography [Title/Abstract] impedance spectroscopy [Title/Abstract] electrical impedance [Title/Abstract]
Cochrane	(dialysis): ti, ab, kw (hemodialysis): ti, ab, kw (peritoneal dialysis): ti, ab, kw (hemodiafiltration): ti, ab, kw (haemofiltration): ti, ab, kw (renal dialysis): ti, ab, kw (extracorporeal dialysis): ti, ab, kw	(bio impedance): ti, ab, kw (electrical impedance): ti, ab, kw (bioelectrical impedance): ti, ab, kw (impedance): ti, ab, kw (resistance electrical): ti, ab, kw (impedance cardiography): ti, ab, kw (impedance spectroscopy)ti, ab, kw
Embase	‘Dialysis’ ‘hemodialysis’ ‘Dialysis extracorporeal’ ‘Extracorporeal dialyses’ ‘Extracorporeal dialysis’ ‘Peritoneal dialysis’ ‘haemofiltration’ ‘hemodiafiltration’ ‘hemodialysis’ ‘Renal dialysis’ ‘Renal dialyses’ ‘Dialysis renal’	‘Impedance’ ‘Impedance spectroscopy’ ‘Impedance cardiography’ ‘Bio impedance’ ‘Bioelectrical impedance’ ‘Impedance bioelectric’ ‘Resistance electrical’ ‘Bioelectrical impedance’

### Inclusion and exclusion criteria

Relevant research included RCTs of patients undergoing regular dialysis treatment where bioelectrical impedance technology (BIS or BIA) was used as a tool supporting treatment, the control group was guided by clinical standards for fluid management, and at least one of the following outcomes was reported: all-cause mortality, left ventricular mass index (LVMI), pulse wave velocity (PWV), and N-terminal brain natriuretic peptide precursor (NT-proBNP). Trials involving patients with amputations, implanted metal stents or pacemakers, pregnancy, severe heart failure, acute complications, malignant tumors, severe malnutrition, chronic liver disease, or chronic obstructive pulmonary disease were excluded from this meta-analysis.

### Literature screening

Two researchers (Kaibi Yang and Shujun Pan) reviewed the literature and applied the inclusion and exclusion criteria by screening the title, followed by the abstract, and then carefully read the full text and extracted data that met the standard (κ = 0.97, *p* = .014). A third-party evaluation was requested if there was any dispute. The following information was collected from the studies: first author, year of publication, country, method of renal replacement therapy, number of patients, follow-up, the primary outcome (all-cause mortality), secondary outcome (LVMI, PWV, NT-proBNP), and BIA method.

### Assessment of risk of bias

We used the components recommended by the Cochrane Collaboration tool to assess the risk of bias in selected trials [13]: allocation concealment, double-blinded trials, and patients lost to follow-up. Each trial was evaluated separately by the two investigators, with a third person settling any disputes.

## Data analysis

The meta-analysis was conducted *via* the RevMan 5.3 software provided by the Cochrane Collaboration. Heterogeneity was assessed using the *I*^2^ test; values above 50% were considered to represent substantial heterogeneity. If the heterogeneity was small (*p* > .1, *I*^2^ <50%), a fixed-effect model was used for the meta-analysis. If the heterogeneity was large (*p* ≤ .1, *I*^2^ >50%) a random-effects model was used for the meta-analysis. Subgroup or sensitivity analysis was used to determine the source of heterogeneity, and subgroup analysis was carried out for the dialysis method and continent. A funnel plot was used to detect publication bias in the literature, and the Egger test was performed using StataSE version 16.0 software to quantify the publication bias of the funnel plot, with *p* < .05 denoting publication bias. The primary outcome of our study was all-cause mortality, which was expressed as a rate ratio (RR). For outcomes reported on a continuous scale, mean differences were used and pooled using a random-effect model in an inverse of variance analysis. All effect measures are presented with a 95% confidence interval (CI). Studies whose original data were displayed as a median and interquartile range were converted to a mean and standard deviation [14], and we extracted the differences between the values at baseline and the end of the study for analysis.

## Results

After the screening, 15 RCTs were included in this meta-analysis (Table 2) [15–29], of which 14 reported mortality [16–25,27–29], 6 reported LVMI [16,18,20,22,26,28], 5 reported PWV [22,23,27–29], and 4 reported NT-proBNP [15,18,23,29]. One study combined lung ultrasound and bioimpedance monitoring [23]. The shortest follow-up time was 12 weeks [15], followed by 6 months [21], 9 months [18], and 1 year (9 studies) [16,17,20,22,24–26,28,29]; 3 trials had a follow-up longer than 1 year [19,23,27].

**Table 2.** Characteristics of included trials.

Source	Participants	Country	n	Technology used	Primary outcomes	secondary outcomes	Duration
Brimble et al. [16]	Regular PD	Canada	65	Bioimpedance using Quadscan 4000 (Bodystat) by the vector graph method assessed every 2 mo	All-cause mortality	LVMI	1 year
Huan-Sheng et al. [25]	Regular HD	China	298	Bioimpedance every month using BCM (Fresenius Medical Care)	All-cause mortality	–	1 year
Hur et al. [28]	Regular HD	Turkey	156	Bioimpedance twice monthly using BCM (Fresenius Medical Care)	All-cause mortality	LVMI, PWV	1 year
Liu et al. [19]	Regular HD	China	445	Bioimpedance every 2 mo using BCM (Fresenius Medical Care)	All-cause mortality	–	13.7 mo
Onofriescu et al. [29]	Regular HD	Romania	135	Bioimpedance every 3 mo using BCM (Fresenius Medical Care)	All-cause mortality	PWV, NT-proBNP	12 mo
Onofriescu et al. [27]	Regular HD	Romania	131	Bioimpedance every 3 mo using BCM (Fresenius Medical Care)	All-cause mortality	PWV	3.5 years
Oh et al. [22]	Regular PD	Korea	137	Bioimpedance using BCM (Fresenius medical care) twice per month	All-cause mortality	PWV, LVMI	1 year
Patel et al. [21]	Regular HD	India	50	Bioimpedance every 15 d using BCM (Fresenius Medical Care)	All-cause mortality	–	6 mo
Paunić et al. [18]	Regular HD	Germany	83	Bioimpedance using BCM (Fresenius medical care) every mo and frequency increased to every wk if OH > 15% or symptoms	All-cause mortality	NT-proBNP, LVMI	9 mo
Siriopol et al. [23]	Regular HD	Romania	250	Monthly lung ultrasound and BIA	All-cause mortality	NT-proBNP PWV	21.3 ± 5.6 mo
Sommerer et al. [15]	Regular HD	Germany	132	Adjust body fluid volume according to BIA	All-cause mortality	NT-proBNP	12 weeks
Tan et al. [24]	Regular PD	China and UK	308	Bioimpedance (vector plot analysis) every 3 mo using BI 101 ASE (Akern, Italy)	All-cause mortality	–	12 mo
Tian et al. [17]	Regular PD	China	240	Bioimpedance every 1–3 mo using	All-cause mortality	–	1 year
Yoon et al. [20]	Regular PD	Korea	201	Bioimpedance every 1–3 mo using BCM (Fresenius Medical Care)	All-cause mortality	LVMI	12 mo
Zhou et al. [26]	Regular HD	Germany	105	Bioimpedance every 3 mo using BCM (Fresenius Medical Care)	–	LVMI	12 mo

HD: hemodialysis; DP: peritoneal dialysis; BIA: bioelectrical impedance analysis; BIS: bioelectrical impedance spectroscopy; BCM: body composition monitor.

### Primary outcome: mortality

**Figure 2.**
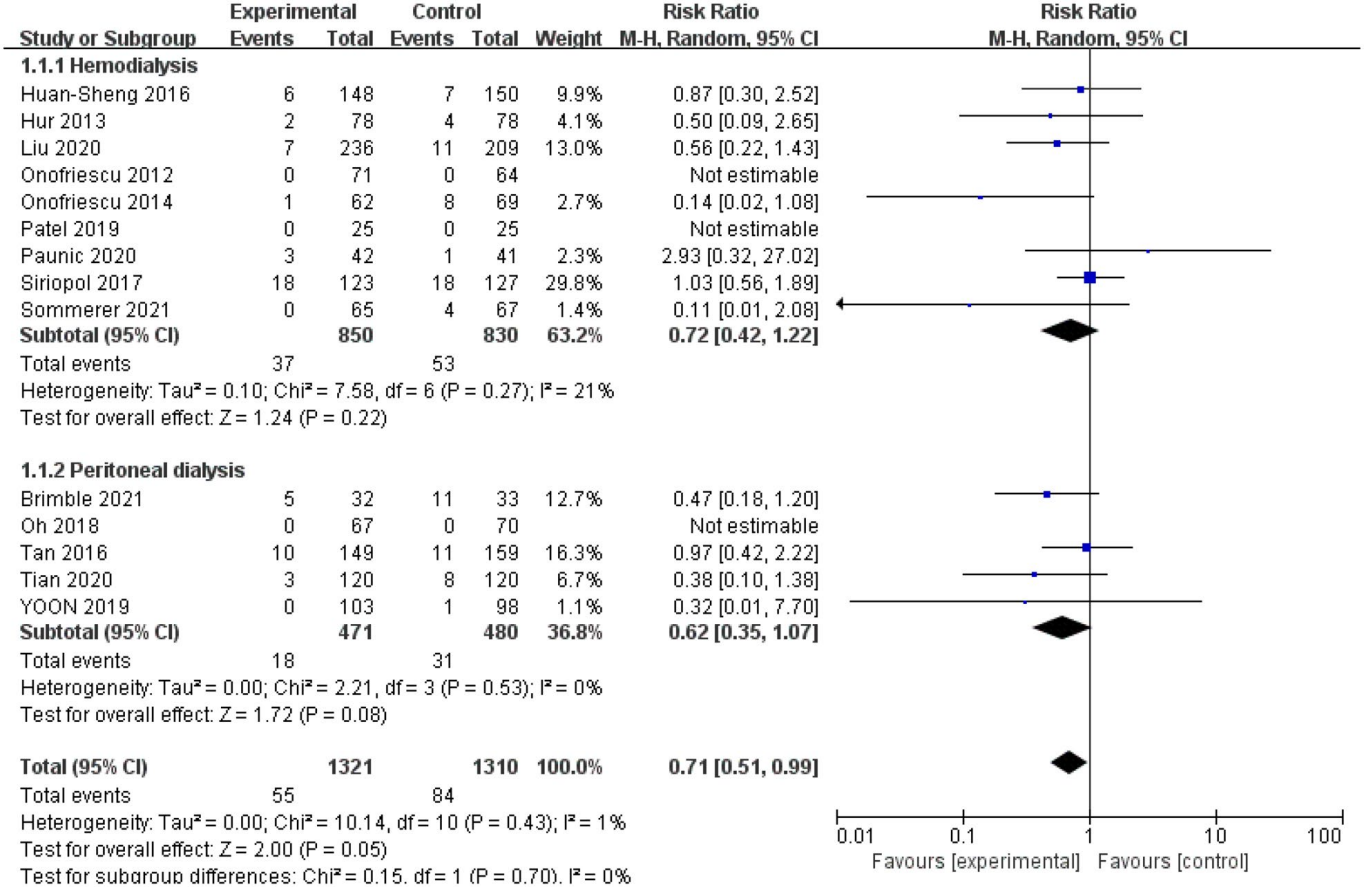
Forest plot of the effect on all-cause mortality in different dialysis groups.

**Figure 3.**
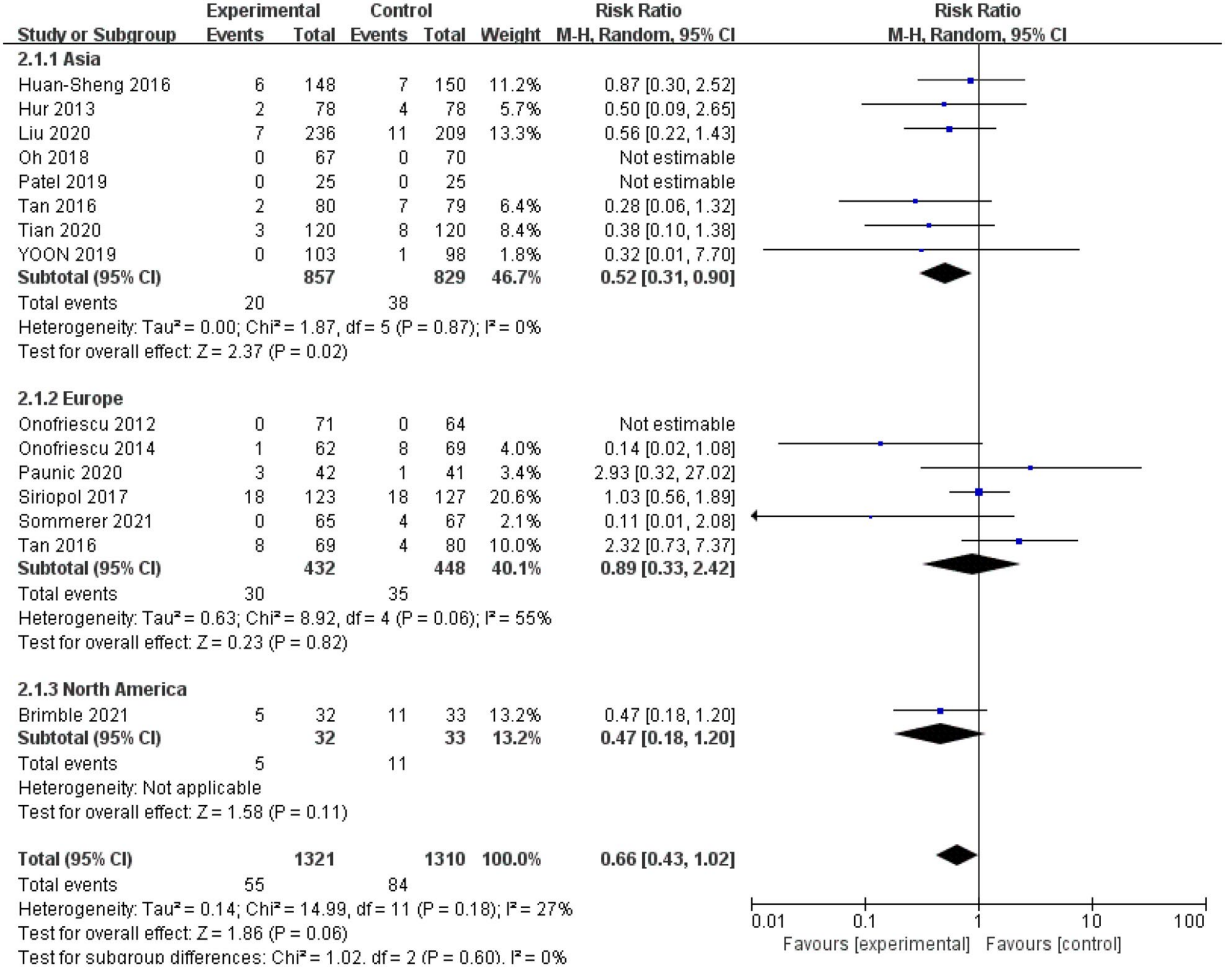
Forest plot of the effect on all-cause mortality in different continents.

The 14 trials with available mortality data enrolled 2631 patients. The random-effect model used for the meta-analysis showed that bioelectrical impedance technology could reduce the mortality of dialysis patients, (RR: 0.71; 95% CI: 0.51, 0.99; *p* = .05; *I*^2^ = 1%). We carried out subgroup analysis according to different dialysis methods and continents (which included Asia, Europe, and North America). Data obtained from the study by Tan et al. [24] that involved 149 patients from the UK and 159 patients from China were divided into Asian and European groups for subgroup analysis. Our subgroup analysis of dialysis methods showed no statistically significant difference in mortality between the intervention and control groups. But subgroup analysis of continents showed the technology could significantly reduce the risk of all-cause mortality in Asian dialysis patients (RR: 0.52; 95% CI: 0.31, 0.90; *p* = .02; *I*^2^ = 0%) (Figures 2 and 3).

### Secondary outcome: LVMI

**Figure 4.**
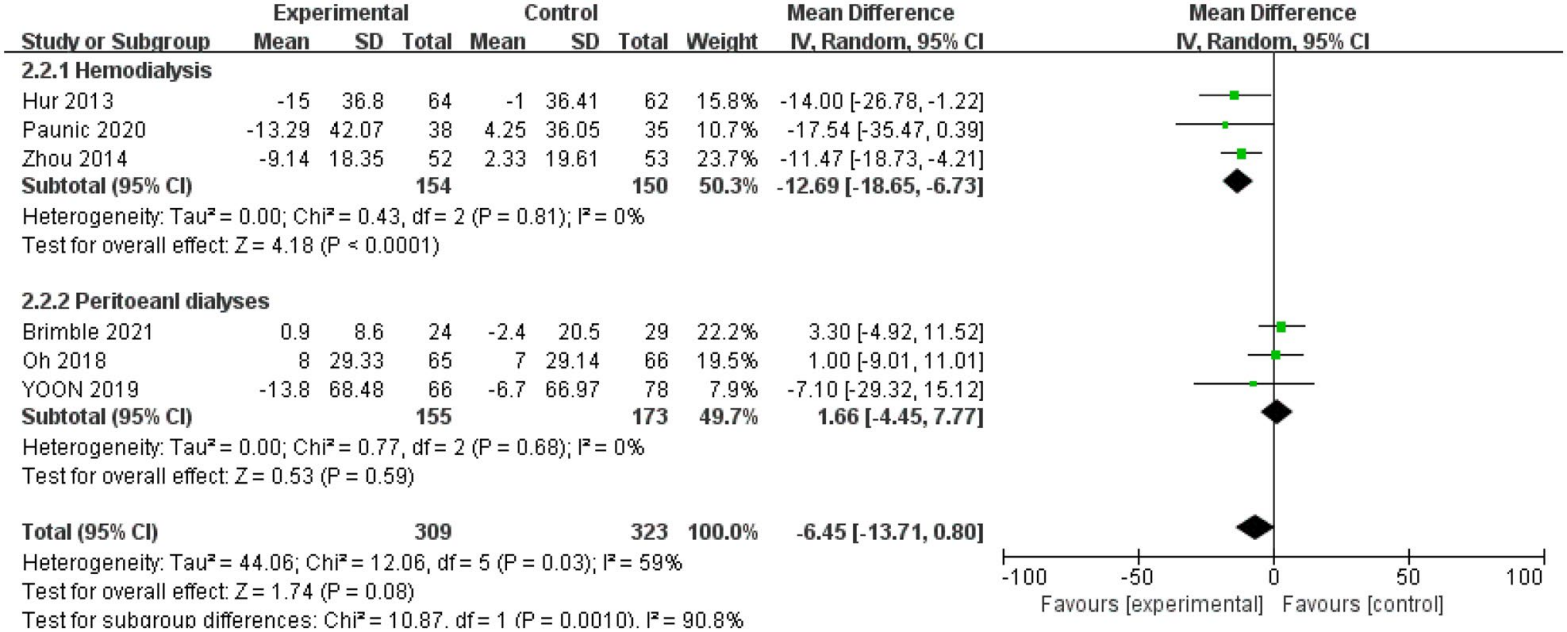
Forest plot of the effect on LVMI.

Six studies [16,18,20,22,26,28] reported data on the LVMI of patients; Brimble et al. [16] measured LVMI by MRI, and others used echocardiography. We extracted data on the difference between the end of the study and baseline levels for analysis. The heterogeneity between studies was large (*I*^2^ = 59%; *p* = .03), so a random-effects model was used for analysis. Meta-analysis results showed no significant difference between the intervention and control groups (MD: −6.45; 95% CI: −13.71, 0.80; *p* = .08). We conducted subgroup analysis according to different dialysis methods. The results showed that LVMI was significantly reduced in the intervention group compared with that in the control group of hemodialysis patients (MD: −12.96; 95% CI: −18.69, −6.73; *p* < .0001; *I*^2^ = 0%), while no significant difference was observed in the peritoneal dialysis group (Figure 4).

### Secondary outcome: NT-ProBNP

**Figure 5.**
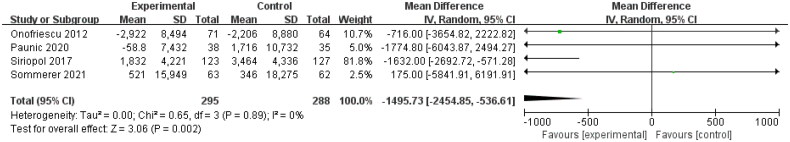
Forest plot of the effect on NT-proBNP.

Four studies [15,18,23,29] provided data on the NT-proBNP level in patients, all of whom were hemodialysis patients. We extracted the data on the difference between the end of the study and the baseline level for analysis. The heterogeneity between studies was small (*I*^2^ = 0%; *p* = .89); therefore, a fixed-effects model was used for meta-analysis. The results showed that the application of bioelectrical impedance technology could significantly improve NT-proBNP (MD: −1495.73; 95% CI −2454.85, −536.61; *p* = .002) (Figure 5).

### Secondary outcome: PWV

**Figure 6.**
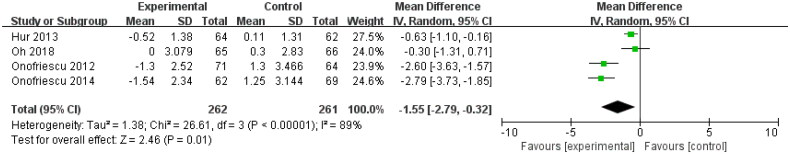
Forest plot of the effect on arterial stiffness (pulse wave velocity in m/s).

Five studies [22,23,27–29] provided data on the PWV of patients. As the heterogeneity among the studies was large (*I*^2^ = 96%, *p* < .00001), a random-effects model was used for the meta-analysis. The results showed no significant difference in the reduction of PWV between the intervention and control groups (MD: −1.00; 95% CI: −2.48, 0.48; *p* = .19). Careful reading revealed that Siriopol et al. [23] used a combination of BIA and pulmonary B-ultrasound as interventions. Therefore, we excluded this article, and the final analysis showed that the intervention group had a significantly more reduced PWV compared with that in the control group (MD: −1.55; 95% CI: −2.79, −0.32; *p* = .01). These results indicated that the intervention of bioelectrical impedance technology can significantly reduce the PWV of dialysis patients (Figure 6). However, the results were not robust due to the large heterogeneity, and the option of using a meta-regression was limited by the number of studies.

**Figure 7.**
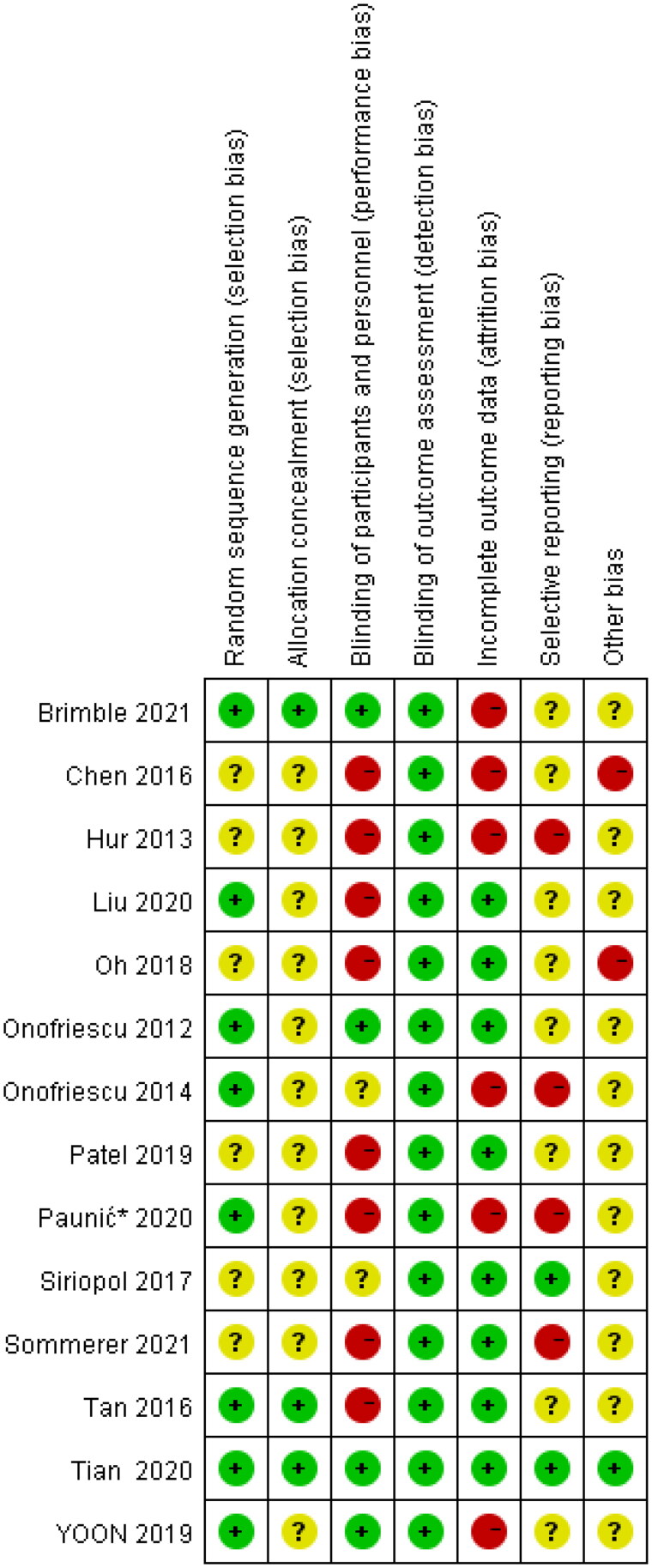
Risk of bias summary graph.

### Risk of bias assessment

**Figure 8.**
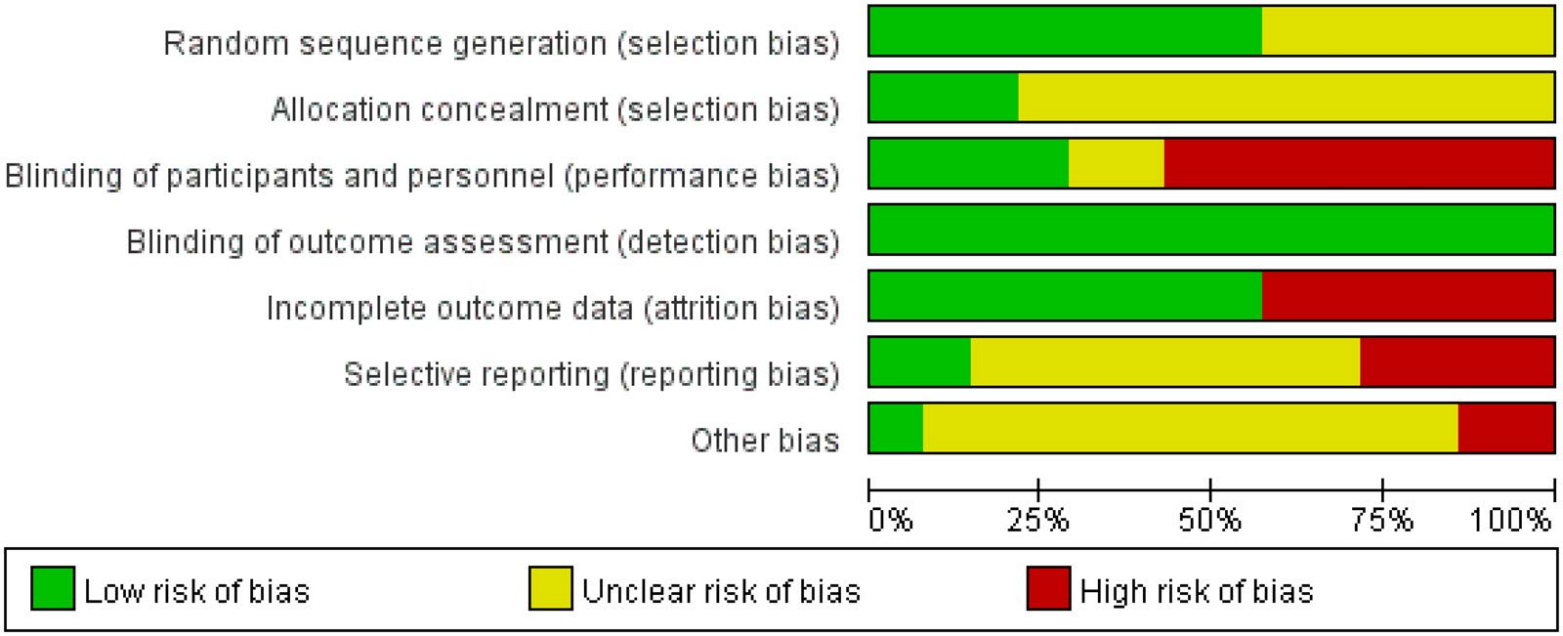
Risk of bias graph.

For trials with all-cause mortality data, 11 had an unclear occult allocation [15,19–23,25–29], 6 were not double-blind [15,18,23,24,27,29], and 6 had a large number of people lost to follow-up [15,17,20,24,25,28]. Thus, the overall assessment showed a moderate bias (Figures 7 and 8).

**Figure 9.**
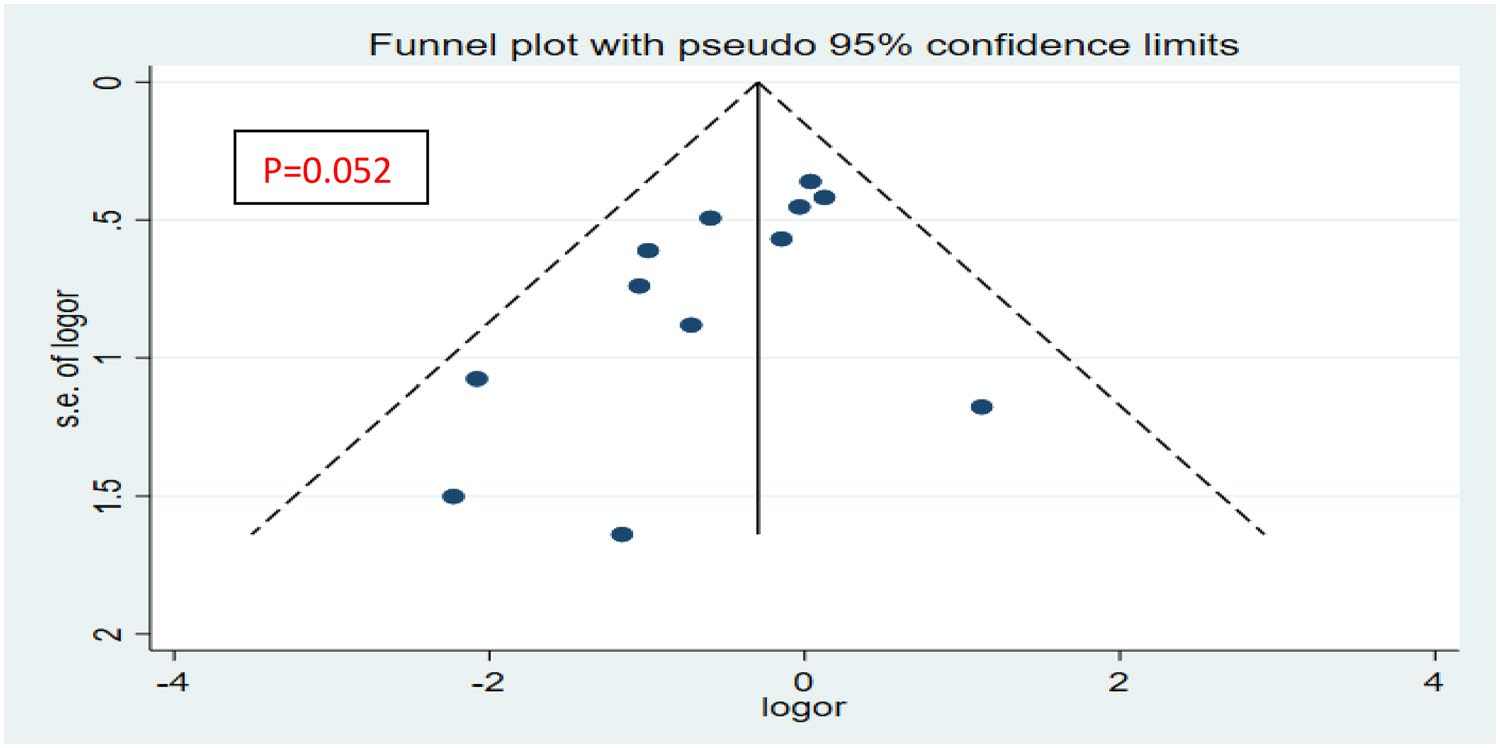
Funnel plot for all-cause mortality.

The funnel plot was used to test the publication bias of the literature included in the main outcome index, and the Egger test was used to quantify whether the funnel plot had publication bias (Figure 9).

## Discussion

The results of this meta-analysis showed that the application of bioelectrical impedance technology to manage dry body weight in dialysis patients could reduce the risk of all-cause mortality, but it could not eliminate the risk. It could decrease NT-proBNP and PWV levels compared with those resulting from the use of clinical standard care. In hemodialysis patients, this intervention also reduced LVMI, which may improve the prognosis of patients.

Fluid overload is highly associated with patient mortality [30]. Clinical auxiliary tools allow clinicians to better manage the dry weight of dialysis patients; however, before applying these tools on a large scale, we must first establish their benefit to patients. The clear criterion is the mortality rate of the patient. The results of our meta-analysis demonstrate that bioelectrical impedance technology could reduce the risk of death of patients. Volume overload has been presumed to be one of the main causes of death in dialysis patients. However, the results of the current three meta-analyses showed that although bioelectrical impedance technology intervention could reduce systolic blood pressure and excessive hydration in dialysis patients, it has no positive effect on their mortality [31–33]. The factors affecting the death of dialysis patients are intricate. Studies have shown that age, diabetes, tumors, smoking, inflammation, residual renal function, and LVH are related to the death of dialysis patients [34,35]. Adjusting the fluid volume in patients with end-stage renal failure to an appropriate ‘dry body weight’ is a slow and persistent process that must be followed up long enough to observe clinically meaningful results. Previous meta-analyses included relatively few RCTs; we included more RCTs with larger sample sizes. Hence, the results are more convincing.

An elevated NT-proBNP level has been associated with the occurrence of cardiovascular events [36] and all-cause mortality in patients with end-stage renal failure [37]. Fluid volume overload can cause ventricular hypertrophy in dialysis patients, and the forced stretching of the myocardium causes more NT-proBNP to be secreted [38]; thus, LVMI and NT-proBNP levels can reflect the patient’s cardiac load. Our analysis showed that bioelectrical impedance technology reduced NT-proBNP levels in dialysis patients and LVMI in hemodialysis patients. The dry weight of hemodialysis patients is adjusted by doctors through analysis of BIA results, while the fluid volume of peritoneal dialysis patients is self-controlled. Since the amount of ultrafiltration in hemodialysis patients may be easier to adjust, the LVMI improvement effect may be more obvious. However, studies on peritoneal dialysis patients have not reported NT-proBNP, which should be addressed in the future.

PWV is an independent predictor of all-cause mortality and cardiovascular event mortality in dialysis patients [39]. An overload of the body fluid volume alters the blood pressure and can lead to an increase in arterial stiffness, which plays an important role in the development of arteriosclerosis in dialysis patients [40]. Patients with end-stage renal disease have arterial wall stiffness before and after dialysis, with an accompanying increase in arterial PWV [41,42]. A study of 1084 dialysis patients from the European Dialysis Center also showed that the risk of death increased by 15% for every 1 m/s increase in carotid-femoral arterial stiffness [43]. An increased fluid volume load especially increases the PWV in peritoneal dialysis patients [44]. Our analysis showed that bioelectrical impedance technology did reduce the PWV in dialysis patients. Our results differ from those of the meta-analysis published in 2017 which had highly heterogeneous results [33]; in contrast, our results were less heterogeneous and more convincing after sensitivity analysis.

Judging whether a new technology is suitable for a large-scale clinical application depends on its advantages and whether it will have fewer side effects compared with traditional methods. Two trials have demonstrated that the use of bioelectrical impedance reduces the occurrence of dialysis hypotension [21,26], while one showed no significant difference [27]. In terms of cardiovascular events, a 3-year trial showed that this technology could reduce the incidence of cardiovascular events in patients [17], while another two trials demonstrated no significant difference [25,26]. One trial showed a lower rate of vascular access thrombosis in the intervention group than in the control group [26]. Overall, relatively few trials report side effects, but we can still conclude that bioelectrical impedance technology has certain advantages over traditional methods.

Our meta-analysis had the following advantages over previous meta-analyses: more studies with larger sample sizes were included, making the results more reliable, and changes in NT-proBNP levels of the dialysis patients were analyzed. This index has not been analyzed in previous studies but can reflect the cardiac function of patients. However, there were some limitations that should be acknowledged. First, some of the included studies had a large number lost to follow-up, and the additional financial pressure on patients associated with using bioelectrical impedance technology was not assessed. In addition, the equipment used for the bioimpedance assessment has variable methods of operation, validated populations for the normal reference range, and accuracies of the results. Thus, there are device-related differences between these randomized trials.

In conclusion, among recipients of maintenance dialysis, the use of bioelectrical impedance adjuncts for fluid management could reduce the risk of all-cause mortality in dialysis patients. It can significantly reduce NT-proBNP levels and PWV in dialysis patients and can also improve the LVMI of hemodialysis patients.

## Data Availability

Data sharing is not applicable to this article as no datasets were generated or analyzed during the current study.
